# Sustainable Compassion Training: Integrating Meditation Theory With Psychological Science

**DOI:** 10.3389/fpsyg.2020.02249

**Published:** 2020-09-11

**Authors:** Paul Condon, John Makransky

**Affiliations:** ^1^Department of Psychology, Southern Oregon University, Ashland, OR, United States; ^2^Department of Theology, Boston College, Chestnut Hill, MA, United States

**Keywords:** empathy, care, compassion, loving kindness, mindfulness, meditation

## Abstract

Meditation programs continue to proliferate in the modern world, with increasing participation from scientists and many others who seek to improve physical, mental, relational, and social flourishing. In developing such programs, the meditation practices have been adapted to meet the needs of modern cultures. However, through that adaptation, important contextual factors of traditional contemplative cultures are often dropped or forgotten. This article presents a system of compassion and mindfulness training, Sustainable Compassion Training (SCT), which is designed to help people cultivate increasingly unconditional, inclusive, and sustainable care for self and others. SCT aims to recover important contextual factors of meditation that flexibly meet the diverse needs of modern secular and religious participants. SCT draws on Tibetan Buddhism in dialogue with caregivers, other contemplative traditions and relevant scientific theories to inform meditative transformation for secular contexts. We provide an overview of SCT meditations that includes both contemplative and scientific theories that draw out important features of them. Each meditation includes novel hypotheses that are generated from this dialogical process. We also provide links to audio-guided meditations.

## Introduction

Meditation continues to gain traction in Western medicine, mental health, and among the general public (Clarke et al., 2018). At the same time, many scholars have expressed concerns about the fidelity of modern meditation programs with the contemplative traditions and cultures that inspired those programs (Sharf, 1995; McMahan, 2008; Harrington and Dunne, 2015; Condon, 2019; Thompson, 2020). Of particular concern, most modern meditation programs have filtered out traditional aspects of meditation that have been situated in communal frameworks of support for broad ethical horizons, because such traditional forms were viewed as too devotional or ritualistic. By deleting those communal dimensions of meditation, a uniquely individualistic, self-help orientation to meditation was newly established in the modern world. Yet patterns of practice that have structured traditional forms of devotion and ritual may be crucial for developing mindful awareness, wisdom, and compassion. Through the lens of attachment theory, we assert that devotional and ritual practices prominent throughout religious traditions—including contemplative forms of Buddhism, Christianity, Islam, Judaism, Hinduism, Confucianism, and many indigenous religions—function to help practitioners develop an unlimited secure base, which they can return to again and again for replenishment, healing, and empowerment (Condon and Makransky, 2020). Through such patterns of practice, these meditators experience themselves and their world as held within the unwavering support of their spiritual benefactors, which empowers them, like their spiritual benefactors, to extend increasingly unconditional, inclusive, and sustainable care to others. We have called this pattern of practice the *relational starting point* for meditation (Condon and Makransky, 2020).

To recover the relational starting point of meditation for secular application in modern contexts, we developed a meditation program called *Sustainable Compassion Training* (SCT)^1^. SCT draws on scientific theories and patterns of contemplative practice from Tibetan Buddhism that are shared with other contemplative traditions, to empower meditative cultivation of increasingly sustaining, unconditional, and inclusive care and compassion (Makransky, 2007, 2011, 2012b, 2019; Lavelle, 2017; Condon and Makransky, 2020; Makransky and Condon, manuscript in preparation). In this article, we present a series of SCT meditation practices and discuss their correlation with theories and findings in modern psychological science. Our aim here is not to validate Buddhist or other religious practices, but rather, to provide a detailed tutorial in an innovative form of contemplative training in compassion, which draws on several major theories of psychology to show how they can newly inform many specific aspects of meditation training for modern people. Scientific theories can be a great support for translating diverse contemplative practices into accessible forms for people who hold modern, scientifically informed worldviews. In addition, dialogue between science and Buddhism can be advanced by inviting in other religious and cultural perspectives (for a similar view, see Thompson, 2020). Here, we provide a description of the theory and practice of the SCT meditations, with links to their audio-guided instructions, to inspire dialogue, hypothesis generation, and research efforts on modern engagement with meditation.

## Overview of Sustainable Compassion Training

Sustainable Compassion Training is a series of meditation practices that have been adapted from three practice traditions of Tibetan Buddhism: Nyingma, Kagyu, and Geluk, informed by dialogue with psychological science and other religious traditions (Makransky, 2007, 2011, 2012b, 2019; Lavelle, 2017; Roeser et al., 2018; Condon and Makransky, 2020; Makransky and Condon, manuscript in preparation). Although SCT was originally adapted from Buddhism, it has been continuously informed by dialogical encounters with participants who work in many fields of service—including healthcare, mental health, education, business, social work, activism, etc.—who represent a diversity of spiritual traditions and scientific perspectives. SCT has been taught in many settings that regularly include people from three different groups: (1) people who work in caring professions or social activism who seek a sustaining power of care and compassion that can empower their lives and work; (2) modern Buddhists who seek reunification with the non-dual, primordial state of awareness that grounds those caring qualities (i.e., nature of mind); and (3) people from other spiritual and religious traditions who wish to deepen their own spiritualities through experiential encounters with Buddhist meditation practices. An emerging fourth category includes scientists who wish to investigate the effects of meditation training and draw on such practices to generate new hypotheses about the mind and human potential. In any given teaching setting, participants typically include members of at least the first three groups, many of whom occupy more than one of those identities. SCT is framed and presented so as to meet the goals of all those participating groups.

**FIGURE 1:**
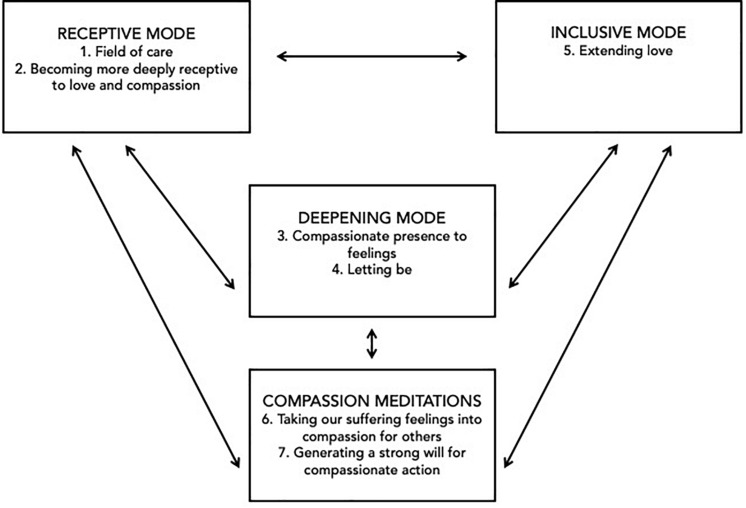
Sustainable Compassion Training Meditations.

Three different modes of contemplative practice form the basis of SCT: (1) receptive mode; (2) deepening mode; and (3) inclusive mode. Each mode includes a set of meditation practices that inform the practices of the other two modes. The receptive mode helps practitioners find new access to hidden qualities of love, compassion, inner safety, acceptance, and wisdom. The deepening mode helps them settle into the source of those qualities in the depth of their awareness—with increasing relaxation, inner peace and spaciousness that is healing and freeing in mind and body. The inclusive mode helps them come from that depth of awareness to respond to others in their deep dignity and potential, with more replenishing, unconditional and expansive powers of care, compassion and discernment for action. On the basis of those three modes of practice, further meditations for cultivating empathy and compassion are introduced, which are designed to help generate compassionate solidarity with others in a sustainable and inclusive way that can avoid empathic distress, compassion fatigue, exhaustion, and burnout (see Figure 1).

There are four key features of SCT that distinguish it from other modern meditation-training programs^2^. First, SCT meets the varied goals of the four groups of participants noted above by constructing an *open secular space* (Makransky, 2012b; Lavelle, 2016). An open secular space explicitly invites practitioners to draw on their own worldviews and religious or scientific backgrounds to inform their meditation practice. SCT accomplishes this by inviting participants to find their own unique way of establishing the relational pattern of practice noted above. This contrasts with the *closed secular space* of many modern secular meditation programs, which avoid discussion of spirituality and religion in favor of creating a singular, scientific framework that everyone must agree to (Gleig, 2019). Of critical import, an open secular space allows practitioners to engage more deeply with the meditation practice than is possible in a closed secular space, because participants are invited to map the pattern of SCT into their own particular worldview, whether religious, scientific or both. For people of diverse religions, the pattern of SCT can map directly into their own religious framework. For non-religious people, the pattern of SCT can map onto scientific frameworks for understanding the relational development of care, as in attachment theory, social baseline theory, and related perspectives (Condon and Makransky, 2020).

The second distinctive feature of SCT is its explicit emphasis on the relational starting point of meditation noted above (Condon and Makransky, 2020). SCT begins not with *self-care* or *extending care*, but with *receiving care* by constructing a relational field in which one is held in the unconditional love of caring connection, benefactors, and/or ancestors. This relational field serves as an outer secure base that evokes the inner secure base of caring capacities that the practitioner needs to extend care reliably to others. This relational starting point of training, by establishing a secure base from which to extend care, mirrors the pattern of emotional development in attachment theory (Cassidy, 2016; Shaver et al., 2016). This pattern of practice also aligns with Social Baseline Theory (Beckes and Coan, 2011; Coan and Sbarra, 2015), which suggests that the presence of supportive others enhances emotion regulatory capacities. The relational pattern of SCT also accords with theories of enactive cognition, which suggest that cognitive capacities are embedded in larger cultural and social contexts (Thompson, 2017). SCT aligns with enactive cognition by embedding its participants in communities of practice that support them in the inevitable challenges they face when attempting to cultivate more unconditional and inclusive compassion for self and others. Practitioners within practice communities can scaffold on mature practitioners who have experienced and overcome similar challenges.

Third, SCT draws on Dzogchen and Mahamudra traditions of Tibetan Buddhism to provide ways for practitioners, empowered by their outer and inner secure base, to settle into the depth of their awareness. This is the deepening mode of SCT, and is accomplished by becoming compassionately present to all feelings and thoughts as a basis for settling into increasingly non-dual states of awareness. These traditions of Tibetan Buddhism, together with Zen traditions of East Asia, suggest that the primordial state of our awareness is a non-dual unity of pervasive openness, pure cognizance, and compassionate capacity^3^. From an attachment theory perspective, we view this kind of awareness as an unlimited secure base (Condon and Makransky, 2020), since it is the primordial source of inexhaustible love and compassion (Makransky, 2007, pp. 33–68; Thondup, 1996; Rinpoche, 2012, pp. 41–80; Varela, 1999, pp. 65–75). This dimension of awareness can function as the ultimate holding environment (Winnicott, 1960; Hoffman, 2015) by providing the unconditional spaciousness and warmth in which patterns of difficult feeling and thought can deeply relax, heal, and release. In accord with this view of primordial awareness, SCT adopts the perspective that caring capacities are an innate feature of the human mind (rather than a skill that must be created). This perspective aligns with some evolutionary perspectives on care and compassion, which suggest they are an innate feature of the human mind that contributed to the survival and flourishing of the human species (Hrdy, 2009; Goetz et al., 2010; de Waal and Preston, 2017; Gilbert, 2019; Marsh, 2019; Tomasello, 2019).

The fourth distinctive feature of SCT is the theoretical view that liberation is available within suffering. This perspective draws on the Mahayana Buddhist position that all emotions are constructed and therefore empty at their core. Liberation occurs with the experiential insight (*vipaśyanā*) of the emptiness of emotions, and the unity of that emptiness with awareness. In this view, “destructive” emotions are distorted expressions of the non-dual awareness that precedes them. Freedom from the so-called destructive emotions, such as greed, hatred, and delusion, is available within the emotions themselves (Ray, 2002, pp. 91–107). This view corresponds with the Theory of Constructed Emotion, which suggests that emotions have no underlying essence (Barrett, 2017). That view can empower the practitioner to be with suffering in an unconditional, spacious way that provides the holding environment for difficult emotions to relax, unwind and release by themselves, i.e., to self-liberate. The self-liberation of emotions appears distinct from meditation programs that foreground self-compassion, which focus on bringing kindness to one’s emotions, rather than simply allowing them to free themselves by revealing their emptiness from within^4^.

In the following sections we describe each mode of SCT in turn. Each mode includes one or two meditations. We present practical instructions for doing each meditation along with scientific theories and findings that help draw out important features of the meditations and inform them. Each meditation includes novel hypotheses that are generated from this dialogical process.

## Receptive Mode

The receptive mode of SCT draws on patterns of devotional practice found in Tibetan Buddhism and other contemplative practice cultures and translates them into newly accessible forms for modern people of diverse backgrounds. Those patterns of practice are mirrored in scientific research findings on attachment priming. In such research, participants are asked to visualize a supportive person, or simply think of concepts like “love,” “care,” “safety.” (Gillath et al., 2005; Mikulincer and Shaver, 2005; Gillath and Karantzas, 2019). Hundreds of studies have shown that such priming can temporarily increase a felt sense of security, even for people with predominantly insecure attachment histories. Most importantly, attachment priming temporarily causes people to offer help to others, have more patience listening to others’ difficult emotions, and have less bias (Mikulincer and Shaver, 2015). This research suggests that attachment priming can be integrated with meditation to recover the relational starting point of contemplative cultures (Condon and Makransky, 2020).

### Meditation 1: Field of Care

A core practice of SCT involves establishing a *field of care*, by identifying and reinhabiting a moment of caring connection that makes one happy to recall, or by bringing to mind an inspiring benefactor or deeply meaningful spiritual figure(s)^5^. To do this, practitioners are provided with examples of diverse examples of caring moments and benefactors, and invited to fill in the content of the meditation in the way that feels most connecting and uplifting for that person. This can be done with a moment from any time in the practitioner’s life when another person was seeing her in her deep worth, was happy to be with her, offered support, listened to her, or wished her well. Or the practice can be engaged by imagining the presence of a benefactor to whom the practitioner feels grateful, or spiritual figure(s) from that practitioner’s own tradition who are inspiring to her. The practice can also be engaged by calling to mind a moment with a pet or a moment in a special place, perhaps in nature, where the practitioner felt deeply well, safe, and at home. Finally, the practice can be carried out by calling to mind a moment of care between others that the practitioner witnessed and that brought joy to her, similar to the concept of moral elevation (Schnall et al., 2010)^6^. The important element is that the field of care evoked by the practitioner genuinely brings joy to or feels uplifting to her, which signifies that it is already providing access to loving qualities from her fundamental awareness. After settling more and more fully into the felt sense of those loving qualities—such as deep acceptance, warmth, being seen as worthy, love, inner safety, joy, etc.—the practitioner is instructed to release the visualization of the field of care, and to relax her mind directly into that felt sense of love, warmth and acceptance. This can help the mind to relax deeply, release its images and mental frameworks, and fall gently, completely open like space. We refer to this final portion of the meditation as the “releasing phase,” which supports deepening trust and unification with the spacious source of the caring qualities in one’s fundamental awareness, from which further such qualities can emerge (for this purpose, all SCT meditations end with the releasing phase)^7^. A guided meditation can be accessed here.

An important feature of receptive mode practices is their consistency with current perspectives on grounded and situated cognition (Barsalou, 2008, 2016). According to theories of grounded cognition, when we recall a memory from our life, it is re-enacted in multiple systems of the brain: motor, visual, kinesthetic, affective, and so forth. During the receptive mode of practice, when practitioners reinhabit a caring moment from their past as happening now, visualizing and simulating its felt sense of security, they are experiencing that moment as fully embodied experience, with all the qualities of care, love, warmth, acceptance, peace, and well-being that come with that.

Following the first receptive mode meditation, it is helpful to name the caring qualities that were experienced during the meditation. By naming felt qualities, such as love, warmth, acceptance, ease, tenderness, safety, feeling seen, gratitude, inner freedom, openness, peace, feeling at home, joy, restfulness, etc., practitioners begin to cultivate an increasing awareness of, and receptivity to, a full spectrum of their innate capacities of care. The capacity to name a full spectrum of caring qualities is analogous to the concept of emotion granularity in affective science, in which labeling emotions with increasing precision and specificity leads to a richer emotional life characterized by greater well-being, discernment, and flexibility around emotional responding (Barrett et al., 2001; Quoidbach et al., 2014; Kashdan et al., 2015; Grossmann et al., 2019). We hypothesize that increasing granularity for caring qualities should lead to an increasing ease with which to notice, access and draw upon such qualities.

### Meditation 2: Becoming More Deeply Receptive to Love and Compassion

Although receptive mode meditations can provide immediate access to inner capacities for care and a felt sense of security (i.e., an inner secure base), this style of meditation can also provoke reactive tendencies associated with relationships from the past. According to attachment theory, each person has a general attachment orientation, but also a hierarchy of attachment representations accumulated from a lifetime of interactions with people in various contexts (Collins and Read, 1994; Shaver et al., 2019). These interactions form the basis for *internal working models*, which include procedural knowledge and script-like expectations for how it is to be in relation with others (Bretherton and Munholland, 2016; Waters and Roisman, 2019). In many cases, these models operate outside of conscious awareness and can include a diversity of implicit messages, including feelings of shame or self-criticism, e.g., “I am not worthy of love” or “If others really knew me, they would not care about me.” Another common working model reinforces a care giving mentality, that dismisses the need for oneself to receive support, such as “I should only focus on caring for others.” Any moment of caring intimacy can re-activate these internal models. For this reason, SCT expands on Meditation 1 by explicitly noticing internal working models and incorporating them into the receptive mode of practice in Meditation 2.

To integrate internal working models into the receptive mode, SCT also borrows from a modern form of psychotherapy, called Internal Family Systems (IFS; Schwartz and Sweezy, 2020). IFS asserts a model of the mind in which people have a multitude of self-representations or “parts of self”—analogous to an array of attachment representations and internal working models in attachment theory. The theory of IFS asserts that these self-representations emerged early in one’s life and took on survival strategies for controlling one’s environment. From an attachment theory lens, these self-representations function as internal working models that lead people to believe that sources of care and love are external and therefore must be controlled. In the case of avoidant attachment scripts, for example, a given internal working model that replays a familiar protective strategy, such as “If I am receptive to care, I will get hurt by others.” These protective strategies can interfere with the receptive mode of practice by shutting down the practitioner’s receptivity to the loving qualities of his own fundamental awareness. However, SCT challenges those beliefs by pointing to the actual source of one’s caring qualities and capacities: the spaciousness, warmth, simplicity, and clarity available in the ground of one’s experience—in one’s fundamental, non-dual awareness. Tibetan scholars and teachers refer to this fundamental awareness as the primordial ground of experience or “the nature of mind” (Makransky, 2007, pp. 33–68; Thondup, 1996; Rinpoche, 2012, pp. 41–80; Varela, 1999, pp. 65–75)^8^. Receptive mode meditations help cultivate an increasing receptivity to, and trust in, caring qualities and their source in one’s fundamental awareness, which undercuts the attachment scripts that assume the source of those qualities lies only in the external world, in other people.

In the second receptive mode meditation, practitioners begin by calling to mind a caring moment or a benefactor as in Meditation 1 to *access* caring qualities from one’s awareness, now with the additional instruction to *notice* any difficulties with the meditation that arise within it and to let the sense of self that is having that difficulty and its feelings be *included* within the compassionate space of those caring qualities. In this way, a space of deep acceptance, care and compassion is provided for all the difficult reactions and feelings that are generated by one’s attachment scripts and triggered by the meditation. That space of care and acceptance allows all such feelings and reactions to relax deeply, settle, and heal in their own time. Any difficulty experienced within the meditation is no longer a distraction, but becomes material to be taken into the process of deepening in practice. As with the first meditation, this meditation concludes with the releasing phase, in which the practitioner releases the visualization and conceptual frameworks, allowing patterns of thought and feeling to unwind and release within the openness, clarity, and warmth of her fundamental awareness. A guided meditation can be accessed here.

Four key hypotheses emerge from Meditation 2. First, the loving qualities experienced when recalling a caring moment or benefactor are enhanced to greater levels of unconditionality through the instruction of the practice. The caring moment or benefactor may be *relatively* unconditional, but the qualities of care that the field of care visualization evokes can become stronger and more unconditional through the instruction to incorporate all self-representations (all “parts”) and feelings that arise into that field of care. Second, by including self-representations within the field of care, the mind learns that it does not have to be identified with any one sense of self. By letting each sense of self be embraced by caring qualities, one’s innate capacities for care and compassion are freed from the restrictive lens of any one self-representation. Instead, the practitioner’s identity shifts from any given self-representation to his larger awareness that holds that self-representation in care and compassion (similar to the hypothesized function of mindfulness to transform self-understanding; Hölzel et al., 2011; Desbordes, 2019). Third, by letting all senses of self and feelings be deeply accepted within the field of care, they can begin to relax, settle, heal and release. Finally, by learning to include any self-representation within a field of care, the practitioner is already learning to extend greater unconditionality to others. We hypothesize that a person’s strengthening ability to let his own self-representations and difficult emotions be held in compassion can strengthen his ability to hold others and their difficult emotions in care and compassion without contributing to compassion fatigue and burnout.

These hypotheses garner support from research on the benefits of cultivating granularity for emotion states. Just as practitioners can cultivate granularity for loving qualities by naming them, so too they can cultivate granularity for various parts of self, i.e., self-representations, with their corresponding attachment scripts. Several empirical findings show that naming feelings with emotion labels can help people become less identified with emotions. Labeling emotionally evocative images, for example, reduces emotional reactivity (Lieberman et al., 2007). Similarly, granularity is associated with greater emotional stability (Hill and Updegraff, 2012; Pond et al., 2012), and less reactivity to social rejection (Kashdan et al., 2014).

Emotional granularity also helps one’s judgments of others become immune from incidental emotion. Incidental emotion refers to a common empirical finding that emotions from one context carry over to other contexts in ways that inappropriately influence subsequent perceptions, judgments, and behavior (Petty et al., 2001). For example, emotional reactions to disgusting images sometimes distort one’s moral judgments of others in a different context (Cameron et al., 2013). Because cultivating emotional granularity supports the ability to dis-identify from emotions, judgments of others become immune from incidental emotion (Cameron et al., 2013). This research suggests that cultivating granularity with senses of self while holding them in a space of acceptance and compassion could support one’s ability to dis-identify or “unblend” from those self-representations, and thereby become more unconditionally present to oneself and to others (Schwartz and Sweezy, 2020).

## Deepening Mode

Receptive mode practices activate innate capacities of care and compassion, by establishing an outer and inner secure base of loving qualities, and by shifting one’s identity from one self-representation to a fuller, more expansive awareness that can hold any sense of self in care. Deepening mode practices of SCT help practitioners increasingly settle into the source of those caring qualities—the utter openness, clarity, and compassionate capacity to be found in the depth of their fundamental awareness (for empirical investigations of non-dual awareness, see Fucci et al., 2018; Schoenberg et al., 2018). According to Dzogchen, Mahamudra, and Zen traditions, this underlying, expansive fundamental awareness is one’s most natural state of being prior to social conditioning. It includes the backdrop of pervasive openness and awareness that is always present behind the narrow focus of one’s conscious attention.

### Meditation 3: Compassionate Presence to Feelings

Intense, stressful aspects of daily life trigger many difficult feelings. People often seek to avoid such feelings by trying to suppress, ignore or distract themselves from them. But when people seek to avoid or suppress feelings, over time, those feelings ironically increase (Wegner et al., 1993; Wegner, 1997), yielding greater sympathetic nervous system reactivity (Gross and Levenson, 1993), which manifests as physical and emotional tightness, including heightened blood pressure, vasoconstriction, and avoidance motivation (Mendes et al., 2003; Mauss and Gross, 2004). In turn, suppression also disrupts interpersonal communication and relationship quality (Butler et al., 2003; Richards et al., 2003). With repetition over time, these physiological and psychological effects of emotional suppression could lead to negative health consequences, including loneliness, hypertension, and coronary artery disease (Uchino et al., 2007; Appleton and Kubzansky, 2014). The consequent tendencies of inner stress and tightness make it difficult to open to the qualities cultivated in all SCT practices—qualities of openness, kindness, compassion, equanimity, and wisdom. One cannot relax and settle more fully into the source of those positive qualities in awareness when one’s mind and body are so caught up in habitual reactions of aversion to feelings. Such stress and tightness also make it difficult to be more fully present to other people in an open-hearted way. These tendencies are exacerbated by many of the systems that shape all of our emotional lives, including our families, communities, educational institutions, and professional environments. Each such kind of community often conveys and reinforces regulation strategies to suppress, avoid, deny, or distract ourselves from emotional feelings (von Scheve, 2012; Thompson, 2014; Menakem, 2017; Brackett, 2019).

All such difficulties with emotional suppression and avoidance suggest a need for innovative practices to process feelings and emotions in a deeply healing way. The first deepening mode meditation, called compassionate presence to feelings^9^, provides a healing, relaxing and releasing way for feelings that arise in one’s life and work to process themselves. This offers a life-giving alternative to the systemic tendency to suppress or distract ourselves from difficult feelings, while further evoking our underlying capacities of care and compassion.

In this meditation, the practitioner learns how to welcome her feelings into a compassionate space where they can relax, settle in their own time, and as needed, heal in their own natural way. “Feelings” here refers to the pleasant, unpleasant, and neutral feeling tones that accompany our physical and mental experiences and to all the emotions with which they are associated. This kind of meditation shows practitioners that they do not have to avoid or suppress their feelings and reactions or habitually act them out. Instead, the practitioner becomes safely aware of feelings in a gentle, deeply allowing way that gives them all the space they need to relax, settle, and find their own place—ultimately a place of inner healing and releasing. Because of their constructed nature, emotional feelings can release and heal in that way if they have the space and freedom to do so. This view builds on constructivist theories of the mind in modern psychology and neuroscience.

The Theory of Constructed Emotion (Barrett, 2017) points to the underlying emptiness of all emotions: emotions are constructed from domain-general capacities (e.g., attention, perception, conceptualization, interoception) in relation to social conditioning without any underlying essence. Although emotions have a relative, social reality, they do not have an ultimate, substantial reality (Barrett, 2012). SCT adds to the Theory of Constructed Emotion by establishing a “holding environment” (i.e., Winnicott, 1960; see also Hoffman, 2015) in which emotions can self-release. By providing a space in which emotions are not elaborated on, ruminated about, or responded to, the process of constructing an emotion can relax and unwind within the space of compassionate awareness, revealing the underlying emptiness and healing property available right within the emotion. This view from the Dzogchen and Mahamudra traditions aligns with research on mindfulness and emotional reactivity, which shows that acceptance is a key ingredient of mindfulness meditation (Lindsay and Creswell, 2017; Lindsay et al., 2018) and allied therapies that relieve stress (Hayes, 2002; Robins et al., 2004).

In this SCT practice of compassionate presence to feelings, the practitioner learns to be more at home with her emotional feelings, even difficult emotions, and they feel safer with her, since they are not being rejected, denied or avoided. This transforms her way of being with others, since her ability to be present to her own feelings safely, with openness and compassion, is what enables her to be present to other people and their feelings in the same way. It is important to highlight that this practice differs from self-compassion: the intention is not to bring extraneous kindness to emotions, but rather, to simply allow them to be, with a space of deep acceptance that lets them free themselves by revealing their emptiness from within. Nothing extra is applied to the emotion.

This meditation has four aspects: (1) notice the feeling within whatever state of mind or body is occurring; (2) allow the feeling to have all the space it needs to find its own place; (3) rest with or within the feeling; (4) then just let everything be, with spaciousness. In the meditation, the practitioner is instructed to become aware first of a physical sensation, then of an emotional feeling by sensing how it feels within one’s body—not just thinking about it in an analytical, disembodied way. The practitioner is guided to become aware of any such physical or emotional feeling in a fully allowing way, gently welcoming it in a spacious, accepting way that lets it settle in its own way. Then, if another physical or emotional feeling replaces the first feeling, the practitioner becomes aware of that sensation in the same deeply allowing, spacious way. A guided meditation can be accessed here.

Several key hypotheses emerge from this compassionate presence to feelings meditation. First, there can be a healing power to this meditation. For a practitioner to be with all of her physical and emotional feelings in such an unconditional way helps her mind and body to relax deeply, unclench, and begin to heal from within. Second, this practice provides the compassionate holding environment that emotions need to reveal their empty nature and further positive qualities of awareness that emerge from that. In compassionate presence practice, emotional feelings are provided a safe space to open into underlying feelings and eventually into the empty core of the feelings, where a deep sense of relief, warmth, inner safety and well-being can be experienced. This profound core of comfort and security, found in the empty essence of feelings, has been called “essence of love,” an aspect of fundamental awareness that provides a secure base from which naturally to love and care for others (Rinpoche, 2012, p. 61). Third, this practice can empower inner steadiness and courage: the process of letting feelings open and heal from within generates equanimity toward feelings and the situations that evoke them. There is a growing awareness that the practitioner does not need to be afraid of the feelings that others trigger in her, which gives her the courage to work with challenging people and circumstances. Fourth, this practice can facilitate compassionate presence to others: the power to be with her own feelings with such compassion and steadiness becomes a power to be with others and their feelings in the same way. Fifth, in the ways noted above, compassionate presence meditation further establishes the core of security that is needed to extend care and compassion to many others in a more reliable, inclusive and unconditional way. This is possible as the practitioner becomes more unconditional and steadfast with regard to her own feelings.

A final key lesson of this meditation is its relevance to each of the other SCT meditations. All of the meditations in SCT introduce a new way of being that deviates from what a practitioner has been used to. This deviation from familiar ways of being—for example, challenging an attachment script that has dismissed all caring moments—gives rise to various emotional feelings and reactions. In this way, all SCT meditations generate the material for compassionate presence to feelings. Whenever any difficulty occurs, the feeling associated with that reaction can prompt the practitioner to do the compassionate presence meditation. And that practice, in turn, accustoms practitioners to become compassionately present to feelings as they arise throughout their days, when not in formal meditation.

### Meditation 4: Letting Be

Each of the prior SCT meditations help practitioners access capacities for care, acceptance, inner safety and compassion from their fundamental awareness that can embrace all perceptions, thoughts, and feelings. In the releasing phase of all those meditations practitioners let the spacious experience of those loving qualities help the mind feel safe enough to relax its grip on contracted frameworks of self and world and relax into the spacious ground of those qualities—a pervasive openness, clarity, and simplicity of awareness beyond all narrow frameworks of mind. In the releasing phase of every SCT meditation, in essence, practitioners settle back into the spaciousness and simplicity of awareness that is always available in the background of their experience.

According to the non-dual perspectives of Dzogchen, Mahamudra, and Zen, there has always been a background of spaciousness in all that we are experiencing, though generally not conscious. The reader may remember a moment when that pervasive background of awareness dawned, perhaps when resting after a long hike while gazing upon a vast sunset sky, or when deeply relaxed and looking panoramically upon the sky over the ocean. In such a moment, a sense of utter openness, simplicity, peace, and clarity may dawn—a totally open, pervasive quality of awareness that was always available in the background but is now brought forward by the panoramic quality of the sky. That is a momentary experience of the pervasive openness and clarity that is always available in the background of experience, but had been hidden by usual habits of thought and reaction.

The next meditation of “letting be” in body, breath, and mind helps the practitioner settle directly into that tranquil, stable background of pervasive openness, simplicity and purity of fundamental awareness. In this meditation, the practitioner is guided to let his awareness settle naturally within the felt experience of body, breath, and mind in sequence, while noticing any feeling of tension, or holding on, within them. Starting with the body, he allows bodily awareness to settle naturally, while letting any sense of holding on within the body relax and settle. The instructions do not suggest he actively try to make anything happen. Instead, they specify a way to let the body draw awareness naturally into increasing unity with itself, as if letting the body do the meditating, while allowing any bodily tension to unwind and settle in its own time, by letting all be. The same process is then carried out with the “letting be” of the breath. In the “letting be” of mind, the practitioner is instructed to notice any grasping within the mind to any mental framework or pattern of thought, and to let that feeling of holding on relax deep within. This gives the mind permission to release its grip on its mental schema and open into the spaciousness available in the background of its awareness, beyond grasping to any framework of thought. The practitioner learns to let that unity of openness and pure cognizance draw his awareness into oneness with it, as if letting it do the meditating. A guided meditation for the three “letting bes” of body, breath and mind can be accessed here.

The first two “letting be-s” draw on the natural power of body and breath to bring the practitioner increasingly into unity with his somatic experience. The third “letting be” of mind helps the practitioner settle into the basic openness, clarity, and simplicity of his fundamental awareness, letting it draw him into increasing unity with it. This third letting be of mind enters the practitioner into a meditative state referred to in Tibetan Buddhist traditions as “tranquil abiding without support” (“tranquil abiding” translates the Sanskrit term *shamatha*). Here the practitioner is not focusing on a particular object or support—like a sound, image, thought or feeling, but settling directly into the basic openness and pure cognizance of his fundamental awareness, no matter what he is perceiving in the foreground. That backdrop of openness and pure cognizance is inherently stable, even though his sense perceptions in the foreground may be shifting and changing. In tranquil abiding without support, the mind learns to settle into the stability of that cognizant background, naturally wide open and radiant.

By learning in that way to abide in the stable backdrop of cognizance and openness, one gains further access to many positive qualities of fundamental awareness: steadiness of mind, stability of attention, inner peace, equanimity, ease of being, joy, with fuller presence to self and others. When the mind is at rest in its stable background of cognizance and openness, it is not caught up in shifting patterns of self-focused reaction, so there is a natural readiness to share, to care, to empathize with, and to love (Rinpoche, 2003, pp. 55–58).

With support of meditation teachers and a community that practices deeply in this direction, a practitioner may also go beyond tranquil abiding as it was described above. When a practitioner is tranquilly abiding on the naturally spacious backdrop of awareness, the experience of subject-object duality is much attenuated, since the mind is resting in itself. But there remains a subtle, subconscious framework of duality—a sense of someone meditating on something, with a little effort to continue to abide in that way. With further progress of practice supported by mature practice community, one’s mind can reach a point that it may open into a more fully non-dual glimpse of its essential nature experienced as a total unity of emptiness, clarity and compassionate capacity—beyond all frames of reference, beyond all dualistic construction, beyond all effort or doing^10^. In that moment, the mind reverts to its most natural, primordial state (Tibetan *nay lug*), which is experienced as a unity of space and pure cognizance that is unimpeded, pervasive, self-cognizant, and self-manifesting. Patterns of thought, feeling and reaction naturally release or “self-liberate” (Tibetan *rang drol*) when this fully non-dual depth of awareness dawns. This non-dual unity of space and pure cognizance provides deepest freedom from identification with self-clinging patterns of thought and reaction, which frees up the innate compassionate capacities of one’s awareness to unfold with greater ease, spontaneity, and inclusiveness. If this thoroughly non-dual recognition of the empty nature of mind dawns, it is typically very brief before dualistic structures of conceptuality and self-representation again draw the mind into identification with them. The training then becomes the instruction to reconnect again with that non-dual recognition in little moments, many times (Rinpoche T, 1998).

Tranquil abiding without support, as described above, provides a stable inner secure base for people in all groups of interest in SCT, including those in service roles and professions who need a secure core of care and awareness from which to become a reliably caring presence to others. The more fully non-dual recognition of the empty nature of mind is often what Buddhists are seeking in SCT—to reunify with their Buddha nature in support of their paths of awakening. This direction of practice through the Letting be meditation into tranquil abiding and non-dual awareness draws from Tibetan Dzogchen and Mahamudra traditions, and can help practitioners in other spiritual traditions explore analogously deep levels of mental settling, centeredness, and inner freedom to love that are part of their own traditions.

## Inclusive Mode

Research in social, cognitive, and developmental psychology reveals the readiness with which the mind engages in categorization that reduces others to a stereotype or limiting judgment. We use the term “reductive impressions” to highlight the pervasiveness of these judgments and the barrier they pose to sustainable and inclusive compassion (Condon and Makransky, 2020). Reductive impressions are a well-known barrier to extending care beyond one’s narrowly identified in-group (Zaki and Cikara, 2015), but reductive impressions also interfere with care for close others. Whenever one loses contact with an inner core of security, appraisals of threat in any context can foster hostile or unfavorable impressions of any person, including close others. A primary purpose of the receptive mode and the deepening mode meditations is to empower and strengthen one’s inner core of security and the corresponding ability to recognize and relate to the fuller humanity in all others (Condon and Makransky, 2020). The inclusive mode meditations aim to make the practitioner more fully conscious of reductive impressions and learn to relate to others beyond those impressions.

There are three features of the earlier meditations that prepare the practitioner in inclusive mode meditation to extend more unconditional, inclusive, and sustainable care. First, when the mind is completely identified with one protective self-representation, perceptions of others are automatically reductive, and one’s capacities for care and compassion become impeded. For example, in a moment when one’s mind is completely identified with a self-representation that is focused on managing things, other people in that moment are reduced just to objects of management. Or if one’s mind is completely identified with a self-representation that is angry at another person, the other is perceived in that moment as just an object of anger, as just bad. This is analogous to research on affective realism, in which affective states bias how a person perceives others (Siegel et al., 2018; Wormwood et al., 2019). Second, when the mind unblends from a given self-representation, by holding that sense of self in compassionate awareness (as in Meditation 2 above in receptive mode), one’s perception of others starts to open, so that others can be sensed in more of their humanity and potential—e.g., sensing them now not just as objects of management or anger, but as fuller human beings who have deep dignity, potential and want to be well and happy just like oneself. Finally, with this opening of perception, one’s capacities of care, love, and compassion become less impeded, so that one can be more compassionately present and responsive to others (Schwartz, 2001, pp. 35–50).

### Meditation 5: Extending Love

To begin the inclusive mode meditation of extending love, the practitioner starts in the receptive mode by accessing caring qualities and energies (as in Meditations 1 and 2 above). She then lets the flow of those energies extend to others, helping her to commune with them—to sense them beyond reductive impressions in their fuller life, dignity, and potential while wishing them deeply well. We use the term “communing” to connote a *preverbal sense of closeness* to another, sensing the other as a subject, a whole life and fuller person beyond superficial impressions and reductive judgments, possessed of great worth and potential.^11^ Communing with others in this way is a kind of “implicit relational knowing,” which refers to non-verbalized forms of communication and affective experiences in relationships (Lyons-Ruth et al., 1998). Both the receptive and inclusive mode meditations construct implicit relational knowing to inform one’s ability to commune with others in a preverbal, affective, embodied manner. Our use of “communing” also corresponds with what the philosopher Martin Buber called I-Thou, rather than I-it—relating to another in their fuller humanity and unconditional worth, rather than as an instrumental means to an end or as an object of dehumanization (Buber, 1970).

In meditation, the practitioner first reinhabits a field of care, through a caring moment or benefactor, and settles into its felt sense of love and care. While continuing to receive the caring energy from their field of care, the practitioner allows that energy to come through her now to whoever is nearby or whoever the practitioner thinks of, infusing their whole being with that loving energy. The practitioner is instructed to let this flow of energy help her commune with others in their deep dignity and worth, to sense others beyond reductive impressions, in their full life experience and potential, while wishing them deeply well. When ready, the practitioner can let the caring energy come through her more broadly to everyone nearby or in widening circles. If the practice becomes too effortful or rigid at any stage, the practitioner can return to the receptive mode (analogous to returning to a secure base as needed for support). The meditation concludes, like all SCT meditations, with a releasing phase that helps the mind relax into the openness, clarity and warmth of its fundamental awareness. A guided meditation can be accessed here.

Three key hypotheses emerge from Meditation 5. First, this meditation helps practitioners experience themselves as an extension of the field of care in which they are held. In essence, the practitioner learns to hold others as she is held and to see others as she is seen in their deep worth and potential. By repeatedly practicing the inclusive mode in daily life, whoever is nearby or comes to mind is included in the practice, which thereby becomes increasingly all-inclusive in a natural way. Second, within this practice, if there is difficulty seeing another person as more than a reductive impression, the mind is identified with a self-representation that is only seeing the other through the lens of that self-representation. When the practitioner notices that, she can settle back into the receptive mode (her secure base), and bring compassionate awareness to that self-representation. When that sense of self begins to feel safer and more at ease, the lens on the other person can open, to sense the other in their fuller life and dignity. This practice introduces a new degree of freedom to choose whether to continue to relate to familiar, often socially conditioned, reductive impressions of others, or to the actual persons beyond those impressions.

The process of unblending from self-representations and reunifying with the openness and clarity of non-dual awareness in the releasing phase can help the practitioner to sense more possibilities in each situation beyond any one lens upon it, with greater space in the mind for innovation, creative responsiveness, and humor (i.e., skillful means, Pye, 2004; Rinpoche, 2012, pp. 211–228). The practitioner can also find greater freedom to take up various roles or self-representations as needed, without being so fully identified with any one of them. In this way, there is greater space and flexibility in the mind to be who or what is needed in the situation.

The loving qualities evoked within the receptive and inclusive mode meditations help the mind to trust and settle into the source of those qualities in one’s fundamental awareness, which is a unity of spaciousness, clarity, and loving capacity. As the mind settles into that expansive awareness in the releasing phase of each meditation and in deepening mode, its patterns of thought and reaction can increasingly unclench and release, which lets the energies and attitudes of love and compassion emerge more freely. In this way, a virtuous cycle of loving power and spacious awareness can increasingly unfold (Makransky, 2007, pp. 87–90; Condon et al., 2019; Thondup, 2015). To promote this synergy of loving capacity and spacious awareness within its three modes of meditation, SCT training encourages ongoing practice of each mode to empower each of the other modes.

Finally, in our view, authentic love and compassion have the discernment to affirm people in their deep dignity while also confronting their harmful behaviors. This set of practices does not involve accepting anyone’s destructive ways of thinking or acting. Instead, it puts the practitioner more fully in touch with others’ humanity and essential dignity. By learning to connect to that dignity and potential in them, the practitioner can challenge people’s harmful ways of thinking and action on behalf of their fuller potential, on their behalf, not only on behalf of others whom their actions may harm (cf. Keller and Pfattheicher, 2013).

## Generating Empathy and Compassion for Action

In SCT, we define compassion as a caring concern for beings that empathizes with them in their suffering and wants to take action to alleviate it. Our definition of compassion includes affective empathy for others’ suffering. This contrasts with a recent claim that compassion which bypasses affective empathy would be more effective and sustainable than compassion with affective empathy (Bloom, 2016). But suppressing affective empathic feelings can lead to ineffective care by making the caregiver less sensitive to others’ felt experience (Sloman et al., 2005; Staton et al., 2007; Haque and Waytz, 2012). Suppressing affective empathy can also lead to negative health and social consequences for the caregiver (Meier et al., 2001). At the same time, however, it must be acknowledged that empathy, as ordinarily experienced, does carry risks of empathic distress, compassion fatigue, and burnout (Williams, 1989; West et al., 2006; Zaki, 2019, pp. 94–118).

Empathic distress occurs when people empathize with others who are suffering and one’s attention turns inward upon oneself, so one gets caught up in the pain of one’s own empathy (Batson et al., 1983; Klimecki et al., 2014). Compassion fatigue occurs when the caring motivation for others shuts down, often because of repeated experience of empathic distress, secondary trauma, or limited efficacy in supporting others (Figley and Figley, 2017). These risks yield a paradox between empathy and compassion: empathy supports effective care for those in need in its sensitivity to what they feel, but can cause burnout for the caregiver. On the other hand, compassion without empathy can reduce the effectiveness of one’s work, and risks personal negative effects stemming from emotional suppression.

To cultivate effective, empathetically informed care, while avoiding empathic distress, SCT proposes that people can make a conscious choice to feel empathy for others in a way that informs and energizes compassion rather than in a way that devolves into empathic distress. This view aligns with motivated choice theories of empathy (Cameron and Payne, 2011; Zaki, 2014, 2019), which assert that empathy is not a limited resource, but rather through a conscious choice, people can feel empathy and expand the scope of their care (Schumann et al., 2014; Cameron et al., 2019). In SCT, we adopt and expand this perspective with the additional resource of the relational starting point, which provides the outer and inner secure base needed to direct one’s empathy for suffering others into compassion rather than empathic distress.

The addition of the relational starting point to support empathic choice aligns with social baseline theory (SBT; Beckes and Coan, 2011; Coan and Sbarra, 2015), which suggests that people serve as a bioenergetic resource that supports emotion regulation for each other. The mere presence of supportive others can reduce one’s reactivity to threat (Coan et al., 2006). The energetic resources made available by others are not limited to their physical presence; reduced threat reactivity can also occur by imagining supportive others as illustrated by research on attachment priming (Mikulincer and Shaver, 2015). A relational starting point of meditation provides the energetic resource and secure base needed to transform the pain of empathy for others who are suffering into empathic concern for them rather than self-involved empathic distress.

An additional concept from health and social psychology, called “reappraisal,” informs SCT compassion meditations. The biopsychosocial (BPS) theory of stress suggests that threat and challenge are two different physiological responses to stress (Blascovich, 2013). *Challenge* is characterized by heightened sympathetic activity, a more efficient cardiovascular profile (e.g., vasodilation in arteries), and stronger approach motivations. *Threat* is characterized by heightened sympathetic activity, HPA, a less efficient cardiovascular profile (e.g., vasoconstriction in arteries), and avoidance motivations (i.e., avoiding challenges, giving up, preparing for defeat). When stress is *reappraised* as helpful, rather than harmful, people experience a physiological *challenge* response rather than a *threat* response, which improves performance and well-being (Jamieson et al., 2016; Borman et al., 2019). Reappraisal is an effective strategy for making use of the physiological energy of stress.

SCT extends the application of *reappraisal* to one’s own suffering and pain of empathy when encountering others’ suffering. First, the practitioner can reappraise his own layers of suffering not as *isolating* himself from the world, but rather as *a source of connection* with all others who share similar layers of suffering. In this way, he learns to experience even his most painful feelings not just as an awful experience but as a precious resource for generating expansive compassion for many beings. Second, in the same meditative process, the practitioner transforms the pain of empathy for suffering beings into an energy of compassion for them. This is a form of reappraisal, akin to reappraising stress as a helpful energy for responding to challenges in the environment (Jamieson et al., 2012).

In this section, we present two meditations adapted from Tibetan Buddhism that employ this application of reappraisal. Within both meditations, the practitioner is guided to make a conscious choice to reappraise the pain of his empathy in such a way as to energize and inform his compassion for many others, instead of getting caught up in empathic distress.

### Meditation 6: Taking Our Suffering Feelings Into Compassion for Others

The first compassion meditation in SCT assumes that the practitioner’s own experiences with suffering can serve as a source of empathic resonance with others’ suffering, which then motivates compassionate responsiveness and action. The establishment of a secure base in meditation practice empowers the practitioner to become more fully conscious of layers of suffering in herself and others, so such suffering can serve as an energetic and informational resource rather than as a cause of empathic distress, fatigue, and burnout. The receptive and deepening mode meditations above help establish and deepen one’s secure base in the protective qualities of love, compassion, and spacious awareness, so one can feel safe enough to permit layers of their own suffering to become more conscious. This reveals similar hidden layers of suffering in everyone else, informing and strengthening one’s empathy and compassion for them.

In Meditation 6, the practitioner consciously allows herself to experience difficult kinds of emotional feeling that she may have previously sought to avoid or suppress, motivated by the altruistic intention to employ them as a fuel for compassion, while supported by her inner secure base of spacious love and compassion (see Table 1 for a list of difficult situations and associated feelings). The practitioner can explore many such feelings as she repeats the meditation, letting repeated daily practice evoke her own list of further difficult emotions to explore. This meditation supports increasing granularity for difficult emotions and suffering feelings, which can inform increasing discernment of others’ analogous kinds of emotion and feeling (Israelashvili et al., 2019).

**TABLE 1:** In Meditation 6, the practitioner is instructed to examine the list below and select one such situation and feeling to explore in the meditation.

• Become conscious of a feeling of physical pain anywhere, or of anxiety that you feel about your body or your health.
• Recall a feeling of not being seen, or of being looked down upon.
• Recall a feeling of strong anger from being betrayed or hurt by what someone did.
• Recall a feeling of intense longing, incompleteness, or addiction.
• Recall a feeling of failure, hopelessness, or despair: “I’m hopeless, unimprovable.”
• Recall a feeling of grief at the loss of a loved one; or grief at the loss of anything such as a job, a relationship, a way of life.
• Recall a feeling of anxiety over meeting all of your obligations and responsibilities, or of attaining enough security for yourself or your family.
• Recall a moment when you were at your *worst*, saying or doing something that makes you ashamed to recall. What feelings come up when recalling that moment?
• Recall feeling lonely, abandoned, or cut off.
• Recall a feeling of fear for a loved one in their vulnerability or mortality.
• Bring to mind fears you have of severe illness, accident, violence, or injury.
• Bring to mind any fears you may feel at your own impending death.

In the meditation, the practitioner begins with the receptive mode by calling to mind and reinhabiting her field of care. While continuing to resonate with a felt sense of care, the practitioner brings to mind a difficult feeling that many others share, like those in the list above, and takes time to experience what it is like to have that feeling, through her own experience of it. To facilitate this exploration, the meditation instructions ask the practitioner to contemplate the following questions: “How does it feel in your heart and mind? How does it feel in your body? What other feelings come up in association with that feeling? How does the whole world look and feel from within this feeling?” After exploring in this way, the practitioner is reminded that many other people experience feelings like this in their own ways, and is guided to sense through her experience how others feel. Then, the practitioner recalls that her whole being is held in unconditional love and compassion from her field of care, and allows all of her suffering feelings to be embraced in that care. In doing so, the practitioner also imagines she is accepting the same powers of love and compassion into everyone else’s analogous feelings, by allowing the caring energy to extend through herself to them all while wishing them deeply well and free of their sufferings. As with prior SCT meditations, the meditation concludes with the releasing phase, allowing all patterns of thought and feeling to unwind within the utter spaciousness, clarity, and warmth of fundamental awareness. A guided meditation can be accessed here^12^.

Several hypotheses emerge from this practice. First, people often get overwhelmed by suffering when it feels like it comprises their whole reality, but this practice prevents that, by helping people experience suffering feelings as encompassed in a larger awareness of compassionate openness, acceptance and warmth, where all such feelings can deeply relax, settle, and heal. Secondly, with that secure base in place, the practitioner can learn to experience her own painful feelings not as isolating her from others but as *connecting* her to others—as a power for compassionate solidarity with them all. The practitioner can increasingly sense everyone around her as possessed of hidden layers of stress and suffering analogous to her own. Thirdly, by sensing hidden layers in all others, including strangers and disliked others, the practice can further break down biases that impede more inclusive and unconditional love and compassion. It can also empower people to be less self-protective, and more ready to listen deeply to others whose culturally, socially, racially, ethnically, or religiously embedded experiences differ from their own. Finally, although this meditative way of accessing capacities of empathy and compassion profoundly supports one’s empathy for others, it is not complete. To more specifically educate empathy, people need to gain perspectives from others (Eyal et al., 2018) through various ways of getting to know each other: connecting with, opening new spaces for people to find their voices, communal activity and activism, literature, film, theater, etc. (Mar and Oatley, 2008; Mar, 2011).

### Meditation 7: Generating a Strong Will of Compassion for Action

Meditation 6 above revealed suffering layers of the human condition that a practitioner shares with many others, reframed one’s own experience of suffering into a basis of compassion for others, and broke down reductive impressions of others by sensing them all as harboring hidden layers of distress and suffering like oneself. Meditation 7, in turn, helps the practitioner become more fully present and responsive to others’ suffering without being overwhelmed by empathic distress or thinking that he has to turn away. Instead, the practitioner can experience his deepening awareness of others’ suffering as a fuel of empathy and compassion that makes him more fully present to them while generating a strong motivation for caring action. Two key purposes, then, of Meditation 7 are: (1) to develop skill at channeling empathy into compassion instead of empathic distress and (2) to bring out a strong will of compassion for action.

As with the inclusive mode meditation and Meditation 6 above, the practitioner begins with the receptive mode by reinhabiting his field of care. While continuing to resonate with a felt sense of care as a secure base, he then brings to mind a person or group whose suffering deeply touches his heart, while sensing the suffering that they must be experiencing. The practitioner is guided in generating empathy for the others’ suffering by asking: How must it feel for them in heart, mind, and body? What other feelings may be arising for them? The practitioner is encouraged to take time to deepen his affective and cognitive empathy in this way. At the same time, the practitioner is explicitly told to avoid getting stuck in the pain of this empathy. Rather, he makes a conscious choice to let the power of this empathy become an intense energy and attitude of compassion that wishes others free of all the pain and suffering that impedes their wellbeing and happiness. The practitioner lets this wish and energy of compassion radiate powerfully from his heart to that person or group, infusing their whole being and environment in its radiant power, wishing them deeply well and free of the causes of their distress and suffering, each in their own best way. After some time, the compassionate wish and energy is extended more expansively to all beings who experience the various sufferings of living and dying, infusing them and their environment in the same radiant power of compassion, wishing them well and free. The meditation concludes with the releasing phase, in which the compassion evoked by the meditation helps the practitioner relax deeply into the openness, clarity and warmth of his fundamental awareness, where all patterns of thought and feeling are permitted to unwind and release. A guided meditation can be accessed here^13^.

Several key hypotheses emerge from Meditation 7. First, as noted, empathic distress occurs when a person’s attention turns inward on himself, so he gets caught up in his own feelings of pain from empathizing with others in their suffering. In contrast, the power of love and compassion in this meditation directs one’s empathic attention compassionately outward toward others, so the practitioner does not internalize the suffering as empathic distress. Second, a further protection from empathic distress includes the wisdom that has been cultivated in all prior meditations of SCT—the awareness that suffering is never the only reality here, but is embraced in a larger reality of openness, warmth and care in which it can transform and deeply heal or release. Finally, the instruction of Meditation 7 also points to a direction of creative responsiveness for action, by turning one’s attention to causes of distress and suffering, encouraging the practitioner to deepen his learning and response to such causes.

## The Continuing Need to Reconnect With a Secure Base in Both SCT and Attachment Theory

Throughout SCT in all facets above, it is not the case that the practitioner leaves behind the receptive mode. The field of care becomes the starting point for all of the inclusive mode and compassion meditations. One never leaves behind the receptive mode—one will need to come back to her secure base recurrently throughout all aspects of the training, to deal with many challenges that arise for anyone learning to become so unconditional toward all one’s feelings and to all other beings, both in their worthiness to be loved and in their layers of suffering. Whenever difficulties arise in any practice or in any aspect of one’s life, receptive mode practices can be re-engaged to reestablish the secure base necessary to proceed.

This enduring connection with receptive mode practice in SCT parallels a lifespan perspective on security within attachment theory. According to attachment theory, one description of security is *autonomy within relatedness*, suggesting that one feels the support from which to explore and navigate the world on one’s own with the confidence that, if one becomes distressed, there is a secure base to return to (e.g., Powell et al., 2013). A core of security includes a strong felt sense of basic safety, comfort with, and curiosity about the world, together with the trust and vulnerability to return the sources of security as needed. Young children are dependent on attachment figures, and gradually, responsive and sensitive caregivers encourage the children’s autonomy. But even so called “secure” adults can have traces of insecurity from diverse relationship contexts, and thus are never fully and always “secure.” They will always continue to depend on support from others. In that way, people with security are able comfortably to navigate back and forth between autonomy and relatedness^14^. This pathway to security parallels that of SCT. Receptive mode practices lead to increasing realization of an unlimited secure base in one’s fundamental awareness and its caring qualities. But the receptive mode practices are never dropped, even among highly advanced meditators in traditional Tibetan culture. In this way, receptive mode practices help the practitioner respond to challenges that arise both in her ongoing practice and in all the challenging circumstances of life.

## Conclusion

The seven meditations of SCT reviewed in this article are designed to give access to innate capacities of love, compassion, equanimity and discernment from the depth of one’s fundamental awareness, capacities that support increasingly unconditional, inclusive, and sustainable care for self and others. This power of care and compassion is sustainable because it draws from an inner secure base of loving qualities and spacious awareness, evoked by a relational field of care, that the practitioner can repeatedly access as needed for replenishment and support. This pattern of practice mirrors both the basic framework for cultivating all-inclusive love and compassion found in many of the world’s spiritual traditions and the kinds of patterns of human emotional development that are described in attachment and social baseline theories. While our perspective needs further empirical investigation, our hypotheses are informed by the deep philosophical and contemplative experiences of long-standing lineages and their intersection with a vast array of theories from modern psychological science, spanning affective, cognitive, social, health, and developmental psychology. SCT meditations and the dialogical approach within this article may be fruitful ground for empirical investigation and ongoing innovations within contemplative practice for modern cultures.

## Author Contributions

Both authors contributed equally to this work and approved it for publication.

## Conflict of Interest

The authors declare that the research was conducted in the absence of any commercial or financial relationships that could be construed as a potential conflict of interest.

## Footnotes

1 SCT has also been called “Innate Compassion Training” (e.g., Makransky, 2011).

2 For a discussion of SCT in comparison with other meditation programs, see Lavelle (2017).

3 Some empirical studies have explored the neural correlates of adept practitioners who have stabilized in such a state of awareness (e.g., Fucci et al., 2018; Schoenberg et al., 2018).

4 Learning to experience feelings in a safe and liberating way does not mean that one must accept the oppressive structures that evoke those feelings. Instead, the practice can give the practitioner more equanimity, courage, discernment, and care with which to challenge those structures (Manuel, 2015; Rogers, 2015; Owens, 2020).

5 The field of care practice is adapted from Tibetan Buddhist practices of refuge and guru yoga (Thondup, 1996, pp. 165–173; Rinpoche, 1996, pp. 29–36, 73–86; Rinpoche, 2009, 146–149). The adaptations presented here make this pattern of practice accessible for a wider public to find a simple, direct entry into the heart of this pattern of practice, and for Buddhists or other spiritual practitioners to help enhance their practice.

6 An additional way to construct a field of care in SCT is to call to mind a moment when the practitioner herself offered care to another. This may be the best starting point for some people who initially have difficulty receiving care. Nevertheless, we hypothesize that moments of receiving care will eventually also need to be engaged for this practice to reach its maximal effect (Condon and Makransky, 2020).

7 The meaning of the term “fundamental awareness” will be further discussed in the next section on the deepening mode of SCT.

8 These terms for the deep nature of conscious experience will be further discussed in the next section on deepening mode.

9 This practice has been adapted from the “Handshake practice” taught by Tsoknyi Rinpoche (Rinpoche, 2012, pp 146–153).

10 Tibetan and Zen Buddhist traditions refer to this empty, non-dual essence of awareness as the deep “nature of mind,” or “Buddha Nature” (Rinpoche T, 1998).

11 We thank Lama Willa Miller for this insightful understanding of communing in the context of this practice.

12 This meditation is adapted from Tibetan *lojong* practices, which are likewise supported by refuge in an unlimited secure base (Rinpoche, 2006; Makransky, 2007; Lewis, 2018; Hur, 2019).

13 This practice draws upon meditations on all realms of suffering in Tibetan cosmologies, which are likewise supported by refuge in an unlimited secure base (see Rinpoche P, 1998; Makransky, 2012a).

14 14We thank Kent Hoffman for sharing his perspective on the meaning of security within attachment theory (Hoffman, personal communication, July 2019).
